# Brain-Derived Neurotrophic Factor Deficiency Restricts Proliferation of Oligodendrocyte Progenitors Following Cuprizone-Induced Demyelination

**DOI:** 10.1177/1759091414566878

**Published:** 2015-01-12

**Authors:** Vladislav Tsiperson, Yangyang Huang, Issa Bagayogo, Yeri Song, Melissa W VonDran, Emanuel DiCicco-Bloom, Cheryl F Dreyfus

**Affiliations:** 1 Department of Neuroscience and Cell Biology, Rutgers Robert Wood Johnson Medical School, Rutgers University, Piscataway, NJ, USA

**Keywords:** BDNF, corpus callosum, cuprizone-induced demyelination, DNA synthesis, oligodendrocyte progenitors, PDGFRα

## Abstract

Brain-derived neurotrophic factor (BDNF) is a member of the neurotrophin family of growth factors that through its neurotrophic tyrosine kinase, receptor, type 2 (TrkB) receptor, increases 5-bromo-2-deoxyuridine incorporation in oligodendrocyte progenitor cells (OPCs) in culture. Roles *in vivo* are less well understood; however, increases in numbers of OPCs are restricted in BDNF+/− mice following cuprizone-elicited demyelination. Here, we investigate whether these blunted increases in OPCs are associated with changes in proliferation. BDNF+/+ and BDNF+/− mice were fed cuprizone-containing or control feed. To assess effects on OPC numbers, platelet-derived growth factor receptor alpha (PDGFRα)+ or NG2+ cells were counted. To monitor DNA synthesis, 5-ethynyl-2′-deoxyuridine (EdU) was injected intraperitoneally and colocalized with PDGFRα+ cells. Alternatively, proliferating cell nuclear antigen (PCNA) was colocalized with PDGFRα or NG2. Labeling indices were determined in the BDNF+/+ and BDNF+/− animals. After 4 or 5 weeks of control feed, BDNF+/− mice exhibit similar numbers of OPCs compared with BDNF+/+ animals. The labeling indices for EdU and PCNA also were not significantly different, suggesting that neither the DNA synthesis phase (S phase) nor the proliferative pool size was different between genotypes. In contrast, when mice were challenged by cuprizone for 4 or 5 weeks, increases in OPCs observed in BDNF+/+ mice were reduced in the BDNF+/− mice. This difference in elevations in cell number was accompanied by decreases in EdU labeling and PCNA labeling without changes in cell death, indicating a reduction in the DNA synthesis and the proliferative pool. Therefore, levels of BDNF influence the proliferation of OPCs resulting from a demyelinating lesion.

## Introduction

In adult animals subjected to a demyelinating lesion, oligodendrocyte progenitor cells (OPCs) derived from cells present in the subventricular zone and the lesion site are able to proliferate and differentiate into myelinating oligodendrocytes (OLGs) (Gensert and Goldman, 1997; Woodruff and Franklin, 1997; Di Bello et al., 1999; Nait-Oumesmar et al., 1999). Similarly, in multiple sclerosis (MS), progenitors increase in areas of active demyelination and remyelination (Wilson et al., 2006). To be in position to influence these OPCs, and potentially enhance the opportunity for remyelination, it is important to identify factors that increase numbers of these cells and define molecular events that regulate this process.

One factor of particular interest is brain-derived neurotrophic factor (BDNF), which is known to stimulate and control OLG development *in vivo* and in culture. For example, BDNF increases numbers of basal forebrain (BF) OPCs in culture (Van’t Veer et al., 2009), and when there is reduced BDNF as occurs in BDNF+/− mice, there are decreases in NG2+ OPCs in the BF. These effects of BDNF extend to differentiation, as BDNF+/− mice exhibit marked reduction in levels of the myelin proteins (including myelin basic protein, myelin-associated glycoprotein, and proteolipid protein in postnatal and adult mice; VonDran et al., 2010). Moreover, BDNF−/− or BDNF+/− mice exhibit decreased numbers of myelinated axons in the postnatal optic nerve, and myelin proteins are decreased throughout the brain of BDNF−/− mice during postnatal development (Cellerino et al., 1997; Djalali et al., 2005; Xiao et al., 2010). At least some of these defects are reversible. For instance, BDNF injection into the ventricles of postnatal day 10 (P10) and P12 mice increases proteolipid protein mRNA in the hippocampus at P14 (Cellerino et al., 1997).

In the present study, we extend observations that indicate that BDNF increases progenitor cell number following demyelination to explore underlying mechanisms. To do so, we took advantage of the cuprizone model. Cuprizone administration into the mouse diet induces OLG cell death and demyelination. However, it also results in a recovery from the demyelination process (Blakemore, 1973a, 1973b; Mason et al., 2001; Matsushima and Morell, 2001), which is associated with increases in OPCs. Previously, it was determined that mice with reduced levels of BDNF exhibit a blunted increase in NG2+ OPCs (VonDran et al., 2011). In the present study, using both platelet-derived growth factor receptor alpha (PDGFRα) as well as NG2+ as markers of OPCs, we explore whether this blunted increase of OPCs is associated with alterations in DNA synthesis and proliferation. Our results indicate that BDNF not only impacts the numbers of OPCs that respond to a demyelinating insult but also influences both the DNA synthesis phase (S phase) and the proliferation of OLG progenitors. The studies indicate that BDNF levels are important for maintenance of the OPC pool that may impact myelin repair.

## Materials and Methods

### Experimental Animals

Breeding pairs of *BDNF^Tm1Jae^* mice on a *129/BalbC/C57* background were previously purchased from Jackson Laboratories (Bar Harbor, ME) and maintained in the Robert Wood Johnson Medical School Animal Facility, which is accredited by the Association for Assessment and Accreditation of Laboratory Animal Care International. Animal maintenance, husbandry, and housing are in compliance with the Laboratory Animal Welfare Act (PL 89-544; PL-91-579). Breeding pairs were maintained by crossing wild-type (WT) and heterozygous animals. The heterozygous mice exhibit approximately 50% of normal levels of BDNF but appear normal (VonDran et al., 2010). The mouse genotype was determined by polymerase chain reaction analysis of ear- or tail-derived DNA as described elsewhere (Ernfors et al., 1994). Mice were housed in a temperature and humidity-controlled environment with a 12-hr light–dark cycle and maintained on standard mouse chow with water *ad libitum* prior to cuprizone treatment.

### Cuprizone Treatment and 5-Ethynyl-2′-Deoxyuridine Injection

At 8 to 10 weeks of age, two BDNF+/+ (WT) and two BDNF+/− littermates were randomly selected for each experiment. One WT or BDNF+/− mouse was fed cuprizone-containing feed, while one WT or BDNF+/− mouse was fed control feed for 4 or 5 weeks. The cuprizone feed consisted of 0.2% cuprizone milled into mouse feed (Harlan Teklad, Madison, WI). The control feed was the same, but without cuprizone supplementation (Harlan Teklad). Food containing cuprizone or control food was changed every 2 to 3 days. In some cases, 5-ethynyl-2′-deoxyuridine (EdU), or its vehicle, saline, was injected intraperitoneally 2 hr before sacrifice.

### Immunohistochemistry

Mice were euthanized by cardiac perfusion with saline followed by 4% paraformaldehyde. The brains were fixed in 4% paraformaldehyde, cryoprotected in 30% sucrose, embedded in Optimal Cutting Temperature compound (OCT), and frozen at −80℃ for immunohistochemistry. Serial 20 µm frozen coronal sections, were prepared and used for analysis.

To identify progenitor cells, sections were stained using an antibody against PDGFRα (Santa Cruz Biotechnology, Dallas, TX) or an antibody against NG2 (EMD Millipore, Temecula, CA) and costained with anti-proliferating cell nuclear antigen (PCNA, a marker for cell proliferation; Santa Cruz Biotechnology) or, in the case of PDGFRα, anti-EdU (Invitrogen, Grand Island, NY).

For PDGFRα and PCNA or EdU costaining, sections were first heated at 95℃ in 0.01 M citrate buffer, blocked in 5% goat serum/0.5% Triton/phosphate buffered saline (PBS) for 1 hr, and then incubated with rabbit PDGFRα primary antibody (1:100, 1 hr at room temperature [RT]). Sections were then washed three times and incubated with anti-rabbit Alexa Fluor 488 antibody (1 hr at RT; Invitrogen). After washing with PBS, sections were incubated with mouse PCNA antibody (1:1000, overnight, 4℃) followed by washing with PBS and incubation with either anti-mouse Alexa Fluor 594 (1:500) or Cy3 antibody (1:300; Invitrogen) for 1 hr at RT. For double immunostaining for PDGFRα and EdU, sections were stained for EdU, according to the manufacturer’s instruction (Click-iT Edu Imaging Kit, Invitrogen).

To stain NG2 and PCNA, sections were blocked in 20% goat serum/1% bovine serum albumin (BSA)/PBS/0.5% triton at RT for 1 hr, incubated with rabbit anti-NG2 (EMD Millipore; 1:500) at 4℃ overnight, then at RT for 1 hr, followed by incubation in Alexa fluor 594 goat anti-rabbit (1:500). Sections were then heated at 95℃ in 0.01 M citrate buffer for 10 min, blocked in 20% goat serum/1% BSA/PBS/0.5% triton for 1 hr at RT, incubated with mouse PCNA antibody (1:1000, overnight, 4℃), followed by incubation with Alexa Fluor 488 goat anti-mouse for 1 hr at RT.

To costain NG2 and PDGFRα, sections were blocked in 20% donkey serum/1% BSA/PBS/0.5% triton at RT for 1 hr, incubated with rabbit anti-NG2 (EMD Millipore; 1:500) at 4℃ overnight, then at RT for 1 hr, followed by incubation in Cy3 donkey anti-rabbit (1:250). Sections were then heated at 95℃ in 0.01 M citrate buffer for 10 min, blocked in 20% donkey serum/1% BSA/PBS/0.5% triton for 1 hr at RT, incubated with goat anti-PDGFRα (R&D, Minneapolis, MN; 1:100, overnight, 4℃), followed by incubation with 488 donkey anti-goat for 1 hr at RT.

For double staining for PDGFRα or CC1 (a marker for mature OLGs) and terminal deoxynucleotidyl transferase dUTP nick end labeling (TUNEL), sections were treated with Cytonin (Invitrogen) for 40 min at RT, and apoptotic cells were determined by assay using the TUNEL Kit, according to the manufacturer’s instruction (TACS 2 TdT-Fluor In Situ Apoptosis Detection Kit, Trevigen, Gaithersburg, MD). Thereafter, sections were stained for PDGFRα as described earlier, while for CC1, sections were blocked in 5% goat serum/0.5% Triton/PBS for 1 hr, and then incubated with anti-mouse CC1 antibody (1:200; Calbiochem, Darmstadt, Germany) for 48 hr at 4℃, followed by washing with PBS and incubation with Cy3 antibody (1:300, 1 hr at RT).

Images were collected using a Leica inverted fluorescence microscope equipped with an Olympus MagnaFire digital camera and ImagePro Plus 7.0 image analysis software. Contrast of whole images was enhanced using Adobe Photoshop.

### Western Blot

To detect PDGFRα protein levels by Western blot, the midline of the corpus callosum was dissected and lyzed. Protein concentration was quantified as previously described (VonDran et al., 2011). Lysates were run on 3% to 8% Tris-acetate gels (Invitrogen) and then transferred to a polyvinylidene difluoride (PVDF) membrane (EMD Millipore). Membranes were blocked in 4% BSA/TBS-T for 1 hr followed by overnight incubation with rabbit anti-PDGFRα (1:750; Santa Cruz Technology) at 4℃. Membranes were then incubated in horseradish peroxidase-linked anti-rabbit antibody (1:3000; GE Healthcare, Little Chalfont, Buckinghamshire, UK). Bands were visualized with a chemiluminescence system (Perkin Elmer, Inc, Waltham, MA). Membranes were stripped and reprobed with anti-β-tubulin (1:2000; Sigma, St Louis, MO) as loading control.

### Quantification and Statistical Analysis

The number of single- and double-positive cells was counted in serial sections taken from the midline of the corpus callosum through the whole extent of the area over the fimbria-fornix, from bregma −0.22 to 0.94 (Paxinos and Franklin, 1997). Magnification for the quantitation used a 40× objective lens. The midline of the corpus callosum (a width of 240 µm) was chosen because of its significant demyelination, as well as its astrogliosis and microglial responses compared with the other regions of the corpus callosum following cuprizone (Schmidt et al., 2013). In total, 36 serial sections were counted for PDGFRα+ cells; of these, 24 sections were assessed for EdU/PDGFRα+ cells from each mouse, and 12 sections from each mouse were assessed for PCNA/PDGFRα+cells. Twelve serial sections were assessed for PCNA/NG2+ cells. Six sections per mouse were used to identify PDGFRα or CC1 cell death by the TUNEL assay. Double counting was eliminated using the Abercrombie correction (Abercrombie, 1946). Statistical analysis was performed with StatView software, and the data were presented as mean ± SEM and analyzed using analysis of variance followed by Fisher’s protected least significant difference post hoc test or the Matched Pair Student’s *t* test as appropriate, and *p* < .05 was considered significant.

For each experiment, one BDNF+/+ mouse fed control feed was compared with one BDNF+/+ mouse fed cuprizone-containing food, one BDNF+/− mouse fed control food, and one BDNF+/− mouse fed cuprizone-containing feed. The labeling index (LI) was defined by the ratio of the numbers of EdU/PDGFRα+ double-labeled cells or numbers of PCNA/PDGFRα+ double-labeled cells over the numbers of total PDGFRα+ cells. Alternatively, the LI was defined by the ratio of the numbers of PCNA/NG2+ double-labeled cells over the numbers of total NG2+ cells. For each individual experiment, the LI of the BDNF+/− mouse fed control feed, the BDNF+/− mouse fed cuprizone, and the BDNF+/+ mouse fed cuprizone was then expressed as percent of the control LI determined for BDNF+/+ mice fed control feed. Within each experiment, then, each group was compared with its own control. Four-week studies were repeated in seven independent experiments. Five-week studies examining PDGFRα + cells were repeated in six independent experiments. Five-week studies examining NG2+ cells were repeated in three independent experiments. TUNEL staining was assessed in four BDNF+/− and four BDNF+/+ mice.

For Western blot studies, BDNF+/+ and BDNF+/− mice fed cuprizone were compared with their own controls (mice fed control feed) for the same time points. Differences between controls and cuprizone-fed mice were then evaluated. Western blots were analyzed using Quantity One V 4.2.1 software (Bio-Rad, Hercules, CA). Statistical analysis was performed with StatView software, and the data were presented as mean ± SEM. Statistical differences were determined using the Student’s *t* test, and *p* < .05 was considered significant.

## Results

### PDGFRα Is Decreased When BDNF+/− Mice Are Compared With BDNF+/+ Mice After Cuprizone

The number of OPCs and their maturation into myelin-producing cells is important for tissue repair and remyelination following demyelination. Because BDNF may be involved in supporting the expansion of oligodendrocyte progenitors, whether reduced levels of BDNF impact the OPC response was examined. To do so in previous work (VonDran et al., 2011), BDNF+/+ and BDNF+/− mice were fed normal or cuprizone-containing feed, and NG2 protein levels were assessed by Western blotting and NG2+ cell numbers by counting. Increases in NG2 protein as well as NG2+ cells were evident at 4 weeks and 5 weeks of cuprizone, but these changes were blunted in the BDNF+/− mice, suggesting that BDNF impacts the response of OPCs to injury. Interestingly, at 6 weeks when increases in OPCs are no longer evident, there was no difference in the BDNF+/+ versus BDNF+/− mice, suggesting that BDNF’s actions are primarily on the cuprizone-elicited increases in OPCs.

**Figure 1.**
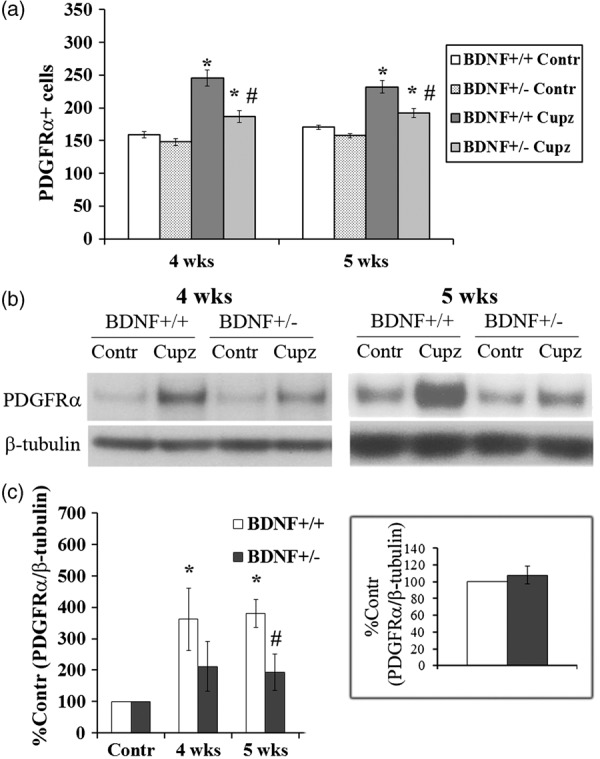
BDNF+/− mice exhibit a blunted increase in PDGFRα + progenitors following cuprizone compared with BDNF+/+ mice. (a) BDNF deficiency does not impact numbers of PDGFRα+ cells under control conditions at 4 or 5 weeks of cuprizone treatment. However, in response to cuprizone, increases in PDGFRα+ cells that occur in the BDNF+/+ mice are blunted in BDNF+/− animals. *Significantly different from control (WT mouse, no cuprizone or BDNF+/− mouse, no cuprizone), *p* < .05, # significantly different from BDNF+/+ mice (fed cuprizone), *p* < .05. The data represent the total number of PDGFRα+ cells quantitated in serial sections taken from the corpus callosum (see “Methods” section). Data were obtained from seven (4 weeks) or six (5 weeks) individual experiments, each containing littermates, either WT or BDNF+/− mice, fed cuprizone or control feed. (b) Western blots of PDGFRα in control mice and animals fed cuprizone for 4 or 5 weeks. Anti-β-tubulin was used to normalize to total proteins levels. (c) The graphs represent densitometric analyses of the blots at 4 or 5 weeks of treatment. Each result is shown as a percentage of its own control (no cuprizone). *Significantly different from control (WT mouse, no cuprizone or BDNF+/− mouse, no cuprizone), *p* < .01, #significantly different from BDNF+/+ mice (fed cuprizone), *p* < .05. Insert represents densitometric analysis of PDGFRa blots of 4- and 5-week-old BDNF+/+ and BDNF+/− unlesioned corpus callosum. Data were analyzed by Student’s *t* test (*N* > 3). BDNF = brain-derived neurotrophic factor; PDGFR=platelet-derived growth factor alpha; WT = wild type.

**Figure 2.**
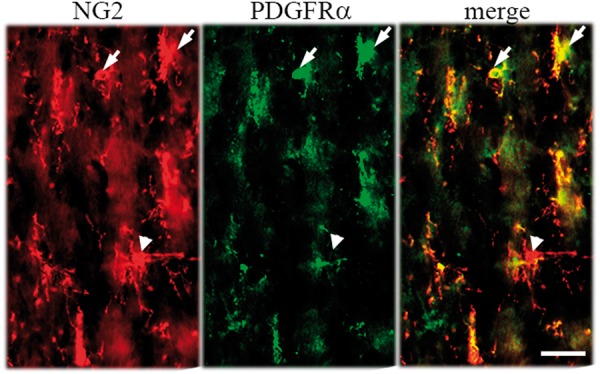
Extensive colocalization (arrows) of NG2 (red) and PDGFRα (green) is evident in the corpus callosum of WT mice, although occasional single-labeled NG2+ cells can be observed (arrowheads). Scale bar = 20 µm. PDGFRα = platelet-derived growth factor alpha; WT = wild type.

To extend these studies and begin defining mechanisms underlying the cuprizone-elicited increases of OPCs, initial studies evaluated PDGFRα, a second marker of progenitor cells. In the absence of cuprizone, numbers of PDGFRα + OPCs in BDNF+/+ mice are not different from those in BDNF+/− animals (Figure 1(a)), suggesting that baseline precursor pools were unaffected by diminished levels of BDNF. However, there is a marked contrast in number of PDGFRα + OPCs following 4 or 5 weeks of cuprizone feed. After cuprizone, the numbers of PDGFRα + OPCs in BDNF+/+ mice increased. While BDNF+/− mice also exhibited an increase in PDGFRa + OPCs after cuprizone, their OPC response is half that of the WT mice. Similarly, levels of PDGFRα protein that increased greater than threefold in the BDNF+/+ mice in response to cuprizone were blunted in the BDNF+/− mice at 4 and 5 weeks (Figure 1(b) and (c)). The blunted responses described here in PDGFRα cells are highly parallel to the previous studies of NG2-labeled cells (VonDran et al., 2011). Consistent with these findings, we detect extensive colocalization of NG2 and PDGFRα in cells within the lesion site (Figure 2). Approximately 80% of the NG2+ population colocalizes with PDGFRα+ cells, while all PDGFRα+ cells are NG2+, suggesting that the markers are identifying essentially the same population of progenitors.

### The Proliferation of OPCs Is Decreased in BDNF+/− Mice Compared With BDNF+/+ Mice After Cuprizone

To begin defining the mechanisms underlying the differential increases in progenitors in BDNF+/− and BDNF+/+ mice, DNA synthesis was assessed by the intraperitoneal injection of EdU 2 hr before sacrifice followed by colabeling with anti-PDGFRα. Incorporation of EdU into DNA identifies the S phase of the cell cycle. Alternatively, PCNA signal was colocalized with PDGFRα+ cells. PCNA is expressed in all phases of the cell cycle and is a marker of proliferation (von Bohlen and Halbach, 2011). Labeling indices (the ratio of double-positive cells to total PDGFRα+ cells) were also assessed.

**Figure 3.**
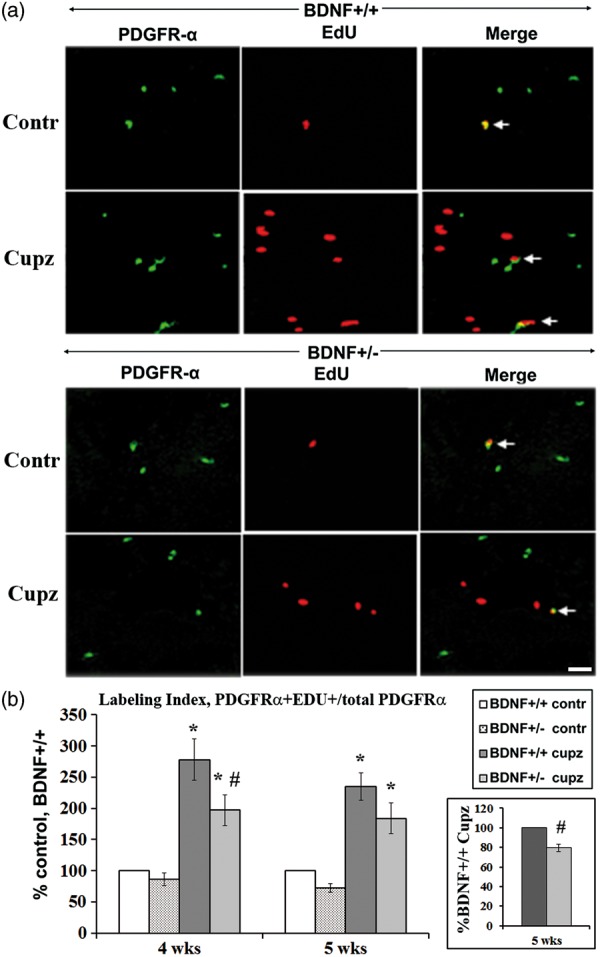
BDNF deficiency reduces enhanced DNA synthesis in OPCs following cuprizone, while no differences are noted in mice fed control feed. (a) PDGFRα/EdU+ cells are indicated by the arrows in sections taken from 5-week control or cuprizone-fed samples. (b) At 4 weeks when labeling indices are expressed as percent control, there is no difference when BDNF+/+ and BDNF+/− mice fed control feed are compared. In contrast, following cuprizone, there are significant differences in the increases in LI in BDNF+/+ mice versus BDNF+/− mice. The LI of the control group (BDNF+/+ mice fed control feed) of the seven independent experiments ranges between 5% and 6%. Results for each experimental animal are presented as a percent relative to its own control for that experiment (see “Methods” section). *Significantly different from control mice (no cuprizone), *p* < .05, #-significantly different from BDNF+/+ mice (fed cuprizone), *p* < .05. At 5 weeks, there is also no difference when BDNF+/+ and BDNF+/− mice fed control feed are compared. Following cuprizone, there are significant increases in the LI of BDNF+/+ and BDNF+/− mice relative to control. *Significantly different from control mice (no cuprizone), analysis of variance, Fisher’s post hoc test. Insert represents the LI of the cuprizone-treated BDNF+/− cells as percent control cuprizone-fed BDNF+/+ cells. #Significantly different from BDNF+/+ mice fed cuprizone, *p* < .05, using the matched pair Student’s *t* test. The LI of the control group (BDNF+/+ mice fed control feed) of the six independent experiments ranges between 5% and 7%. Results for each experimental animal are presented as a percent relative to its own control for that experiment (see “Methods” section). Scale bar = 20 µm. BDNF = brain-derived neurotrophic factor; PDGFRα=platelet-derived growth factor alpha; OPCs = oligodendrocyte progenitor cells; EdU+ = 5-ethynyl-2′-deoxyuridine; LI = labeling index.

We speculated that the diminished oligodendrocyte progenitor response after cuprizone in the BDNF+/− mouse might depend on deficits in DNA synthesis and/or proliferation. To examine this issue, we first examined S phase entry in the different genotypes at 4 and 5 weeks of cuprizone (Figure 3). At both time points, a subset of PDGFRα+ cells was labeled with EdU in both the BDNF+/+ and +/− mice, fed control or cuprizone-containing food (Figure 3(a)). When the LI was assessed in BDNF+/+ and BDNF+/− mice fed control feed, there was no difference in baseline LI, suggesting that altered BDNF levels do not impact the overall rate of DNA synthesis in the PDGFRα+ precursor pool under normal conditions (Figure 3(b)). However, when challenged by cuprizone, the BDNF+/+ mice exhibited a greater than twofold increase in mitotic LI relative to animals fed control feed, indicative of a robust increase in cells synthesizing DNA. This suggests the observed increase in total precursors depended on enhanced proliferation that followed cell cycle progression. In contrast, in the BDNF+/− mice, there was a blunted increase in the mitotic LI, compared with the BDNF+/+ response, indicating that reduced BDNF limited significantly the ability of OPCs to enter the cell cycle.

**Figure 4.**
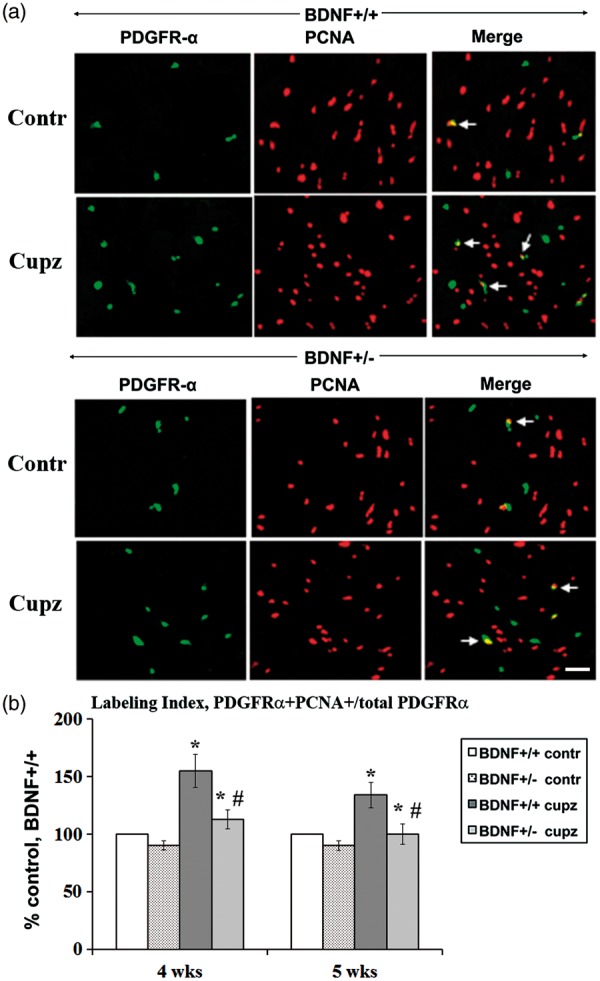
BDNF deficiency at 4 or 5 weeks reduces enhanced proliferation in OPCs following cuprizone, while no differences are noted in mice fed control feed. (a) PDGFRα/PCNA+ cells are indicated by the arrows in sections taken from mice fed control or cuprizone feed for 5 weeks. (b) When the labeling indices within individual experiments are expressed as percent control, there is no difference when BDNF+/+ and BDNF+/− mice are compared at either time point. In contrast following cuprizone, there are significant differences in the increases in LI in BDNF+/+ mice versus BDNF+/− mice. The LI of the control groups (BDNF+/+ mice fed control feed) for the seven independent experiments ranges between 11% and 17% at 4 weeks and for the six independent experiments ranged between 21% and 25% at 5 weeks. Each group value is expressed as a percent relative to own control for that experiment (see “Methods” section). *Significantly different from control (no cuprizone) mice, *p* < .05. #-Significantly different from BDNF+/+ (fed cuprizone) mice, *p* < .05. Scale bar = 20µm. BDNF = brain-derived neurotrophic factor; OPCs = oligodendrocyte progenitor cells; PDGFRα = platelet-derived growth factor alpha; LI = labeling index; PCNA = proliferating cell nuclear antigen.

One possible mechanism underlying reduced S phase entry would be a diminished overall proliferative precursor pool in the BDNF+/− mice. To define this population, we performed double immunolabeling, identifying PDGFRα+ precursors that coexpress PCNA (Figure 4). As was the case in PDGFRα/EdU labeling, a subset of PDGFRα+ cells expresses PCNA at 4 and 5 weeks of cuprizone feeding (Figure 4(b)). Moreover, under conditions of normal feed, the baseline PDGFRα/PCNA double-labeled cells were not different between genotypes, a result consistent with no genotype differences in the mitotic LI (Figure 3). However, after cuprizone treatment, BDNF+/+ mice exhibited a significant increase in PDGFRα/PCNA double-labeled cells compared with controls, whereas BDNF+/− mice exhibited a significant blunting of the effect (Figure 4(b)). In aggregate, the data suggest that when BDNF is limited, the oligodendrocyte precursor pool and its proliferation are reduced.

## NG2+ Cells Act Similarly to the PDGFRα+ Population

**Figure 5.**
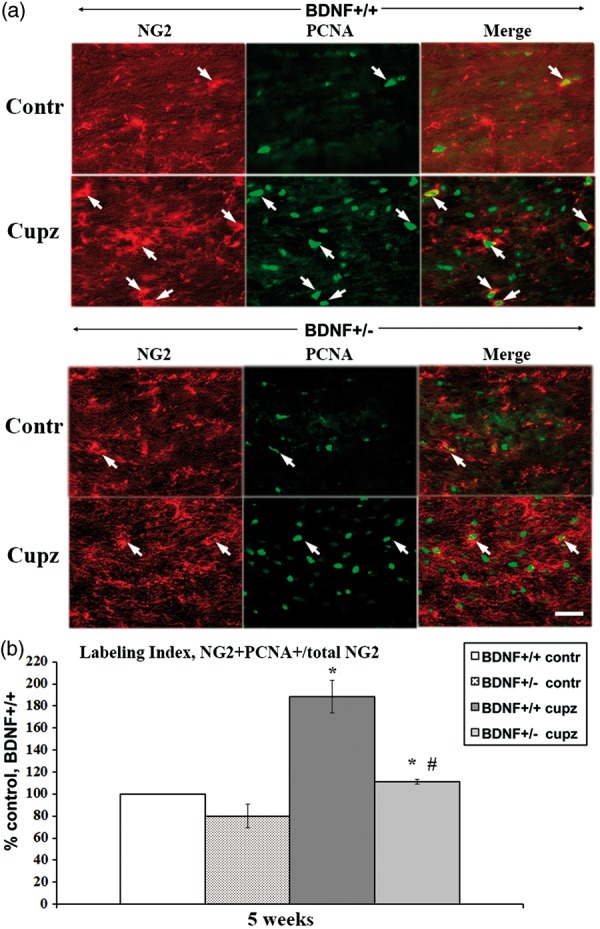
BDNF deficiency at 5 weeks reduces enhanced proliferation in NG2+ OPCs following cuprizone, while no differences are noted in mice fed control feed. (a) NG2/PCNA+ cells are indicated by the arrows in sections taken from mice fed control or cuprizone feed for 5 weeks. (b) When the labeling indices within individual experiments are expressed as percent control, there is no difference when BDNF+/+ and BDNF+/− mice are compared. In contrast following cuprizone, there are significant differences in the increases in LI in BDNF+/+ mice versus BDNF+/− mice. The LI of the control groups (BDNF+/+ mice fed control feed) for the three independent experiments ranges between 15% and 21%. Each group value is expressed as a percent relative to own control for that experiment (see “Methods” section). *Significantly different from control (no cuprizone) mice, *p* < .05. #-Significantly different from BDNF+/+ (fed cuprizone) mice, *p* < .05, *n* = 3 independent experiments. Scale bar = 20µm. BDNF = brain-derived neurotrophic factor; OPCs = oligodendrocyte progenitor cells; PCNA = proliferating cell nuclear antigen; LI = labeling index.

NG2 and PDGFRα are largely colocalized within the lesion site (Figure 2), suggesting that they are labeling essentially the same population of progenitors. However, to confirm that the NG2+ cells respond, as do the PDGFRa progenitors, with a reduction in proliferation in mice with reduced levels of BDNF, we performed studies to evaluate the NG2+/PCNA+ population at 5 weeks of cuprizone. As was the case with PDGFRα, a subset of NG2+ cells expresses PCNA in mice fed control feed (Figure 5(a)). However, after cuprizone treatment, increases in NG2+/PCNA+ cells seen in BDNF+/+ mice were reduced in the BDNF+/− mice (Figure 5(b)). The data indicate that reduced levels of BDNF impact both NG2+ and PDGFRa+ progenitors in parallel fashion.

### Cuprizone Is not Toxic for OPCs but Is Toxic for Mature OLGs

**Figure 6.**
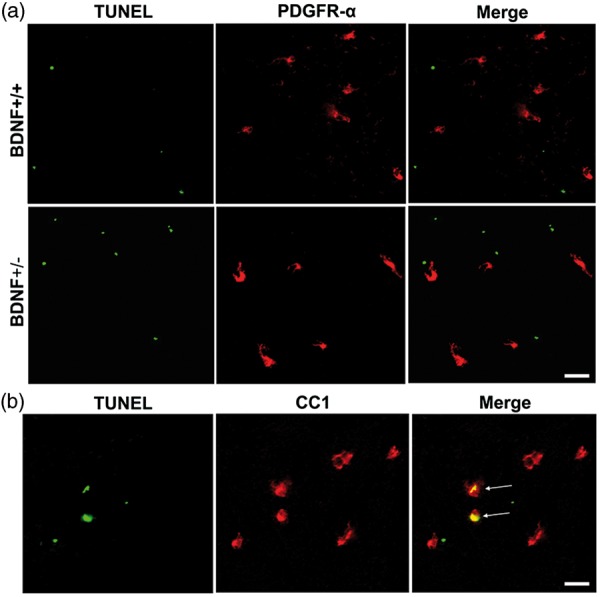
PDGFRα+ cells do not undergo apoptosis after cuprizone toxicity in either BDNF+/+ or BDNF+/− mice as detected by PDGFRα/TUNEL assay. Oligodendrocyte apoptosis is detected in mice following cuprizone toxicity. TUNEL staining indicated in green and is not associated with PDGFRα+ cells, indicated in red (a). TUNEL staining is associated with CC1+ cells indicated in red (b). Arrows indicate double labeling. Six sections per mouse were used to identify PDGFRa or CC1 cell death by the TUNEL assay. Four BDNF+/+ mice and BDNF+/− mice were sampled. Scale bar = 20µm. PDGFRα = platelet-derived growth factor alpha; BDNF = brain-derived neurotrophic factor; TUNEL = terminal deoxynucleotidyl transferase dUTP nick end labeling.

While the foregoing data suggest that OLG proliferation is diminished when BDNF is limited, an alternative mechanism may also involve programmed cell death. To determine whether the reduction in numbers of progenitors in the BDNF+/− mice may be affiliated with cell death, we examined apoptosis using PDGFRα/TUNEL double immunostaining in BDNF+/+ and BDNF+/− mice following 4 weeks cuprizone treatment. No colocalization of PDGFRα+ cells with TUNEL was detected (Figure 6(a)). This suggests that proliferation differences were not due to cell death at this time because TUNEL labeling of PDGFRα cells was not seen in either genotype. On the other hand, examination of mature CC1+ OLGs, known to die during this time period (i.e., Arnett et al., 2001; Bénardais et al., 2013), did colabel with TUNEL (Figure 6(b)), indicating the sensitivity of the assay.

## Discussion

The present study indicates that following a cuprizone-elicited demyelinating lesion, WT BDNF+/+ mice exhibit an increase in PDGFRα+ progenitors that is due to increased DNA synthesis and cell proliferation. On the other hand, when BDNF is limited as occurs in BDNF+/− mice,the increase in PDGFRα+ progenitors is reduced, a deficiency that depends on reductions in both DNA synthesis and subsequent cell proliferation. Similar effects are noted when NG2+ cells are evaluated. This effect is not due to differential actions on cell death.

This new work confirms and extends previous observations that indicated that in culture BF OPCs respond to BDNF by increasing DNA synthesis (Van’t Veer et al., 2009) and differentiation (Du et al., 2006). The increase in DNA synthesis observed in culture, suggesting levels of BDNF regulate cell proliferation, is consistent with *in vivo* studies in which BDNF+/− mice exhibit decreased numbers of NG2+ progenitor cells at postnatal stages and in adults (VonDran et al., 2010) and after a demyelinating lesion elicited by cuprizone treatment (VonDran et al., 2011). The present work now proposes a mediating mechanism that underlies the previous *in vivo* work.

## Effects of BDNF on OPCs Are no Longer Evident after 6 Weeks of Cuprizone Treatment

In previous work, it was found that increases in progenitors, evident at 4 and 5 weeks of cuprizone, are no longer observed at 6 weeks. Moreover, differences in the progenitors evident in the BDNF+/− and BDNF+/+ populations are also gone. Interestingly, the absence of differences in cell numbers also pertains to the postmitotic OLG lineage cells evident in the lesion site, as the numbers of CC1+ OLGs are not different in the BDNF+/+ and +/− mice (VonDran et al., 2011). This suggests that the differential effects on proliferation do not impact the final number of cells that go on to express mature traits. (However, note that although the numbers of OLGs are similar, the cells maturing in an environment deficient in BDNF are limited in their ability to produce normal levels of myelin proteins; VonDran et al., 2011.) These results are reminiscent of previous studies examining the BF during development and in the adult in which BDNF+/− mice exhibit reduced numbers of NG2+ cells in the BF, but similar numbers of postmitotic CC1+ cells that were reduced in their ability to produce normal levels of myelin proteins (VonDran et al., 2010).

It was suggested in these previous studies that it may only be a subpopulation of the progenitors that go on to mature into postmitotic OLG populations, and the other progenitors might die. In the present work, we evaluated TUNEL staining at 4 weeks, but we cannot rule out the possibility that differential death of the progenitors is occurring between 5 and 6 weeks following cuprizone. Such an observation would be consistent with studies, indicating that 50% of OLG lineage cells undergo cell death (Barres et al., 1992) during development and in particular during early myelination (Trapp et al., 1997). Alternatively, it has been reported that following a lesion, a subset of OPCs, known to predominantly mature into OLGs (Zuo and Nishiyama, 2013), may, surprisingly, mature into Schwann cells (Zawadzka et al., 2010). Based on these reports, it is possible that BDNF impacts either the death or the fate of a subset of OPCs that does not go on to become mature OLGs, subjects for future investigation.

## The Significance of the Differential Increase in Progenitors in BDNF+/+ Versus BDNF+/− Mice

If differences in numbers of OLG lineage cells are gone by 6 weeks, what then is the importance of the effects of BDNF on progenitor cell numbers and can it apply to other progenitor populations? The literature suggests that the NG2 population of cells may serve roles other than being progenitors that mature into OLGs. Thus, effects on final OLG cell numbers may not be the only important outcome. For example, NG2+ cells within the hippocampus, cortex, cerebellum, optic nerve, and corpus callosum are known to receive glutamatergic synaptic contacts (Lin and Bergles, 2004; Lin et al., 2005; Kukley et al., 2007; Ziskin et al., 2007) and respond to glutamate by induction of calcium-dependent phosphorylation of extracellular signal-regulated kinase (ERK1/2) (Hamilton et al., 2009). Moreover, in other work, NG2+ cells are known to receive Gamma-Amino Butyric Acid (GABA) ergic synapses and respond to GABA stimulation by increasing BDNF (Tanaka et al., 2009). These cells are also reported to exhibit processes that are directed between axon terminals (Ong and Levine, 1999) and extend to nodes of Ranvier (Butt et al., 1999). The possible participation of NG2+ cells in a synaptic network may allow them to modulate neural signals within the recovering brain and potentially influence the lesion environment. It is intriguing to hypothesize that BDNF may influence these NG2+ cell properties to influence the environment of a demyelinating lesion, a direction to pursue in other studies.

## Effects of BDNF on Increases in Cell Number Are not Limited to OPCs

These results indicating that BDNF can increase numbers of OPCs are consistent with studies of McTigue et al. (1998) who found that BDNF elevates numbers of proliferating OLG lineage cells after spinal cord injury and are reminiscent of BDNF effects on neuronal progenitors. For example, BDNF administration into the lateral ventricle of adult rats results in significant increases in numbers of newly generated neurons in the adult olfactory bulb, the striatum, septum, thalamus, and hypothalamus (Zigova et al., 1998; Pencea et al., 2001). When BDNF is injected into the hippocampus, increases in proliferating granule cells are observed (Scharfman et al., 2005). BDNF associated with human bone marrow-derived mesenchymal stem cells enhances numbers of subventricular neural stem cells in a stroke model (Jeong et al., 2014). These increases in progenitors may be due to enhanced DNA synthesis and cell proliferation. Thus, VGF, a factor that acts downstream of BDNF, enhances both of these processes in hippocampal progenitor cells *in vitro* and *in vivo* (Thakker-Varia et al., 2007). Taken together, the studies suggest that BDNF may play a general role in multiple progenitor populations in the mature brain and after injury.

## Roles of Other Growth Factors on Progenitors

Our study that confirms effects of BDNF on dividing cells following a demyelinating lesion adds BDNF to a growing list of growth factors that can influence the progenitor population. For example, following cuprizone-elicited demyelination, proliferation is inhibited in mice lacking insulin-like growth factor1 receptor (IGFR1), or tumor necrosis factor-alpha, or tumor necrosis factor receptor-2 (Arnett et al., 2001; Mason et al., 2003). Similarly, mice with decreased levels of PDGFRα exhibit decreases in DNA synthesis following cuprizone (Murtie et al., 2005). Consistent with these reports of decreases in factors inhibiting proliferation are those indicating that addition of growth factors may increase the proliferative population. Thus, intracranial administration of a combination of the growth factors PDGF-AA, basic fibroblast growth factor, neurotrophic factor 3, and insulin-like growth factor1 resulted in increases of NG2 cells and numbers of NG2/5-bromo-2-deoxyuridine (BrdU)+ cells in the corpus callosum (Kumar et al., 2007). We suggest that BDNF in combination with these factors may further enhance numbers of progenitor cells and that it may do so by enhancing proliferation and DNA synthesis.

## Significance of Increases in BDNF to Disease

In MS, there is a correlation between BDNF and the severity of MS symptoms. MS patients exhibit decreased levels of BDNF in serum and cerebrospinal fluid during disease activity. Moreover, after treatment with interferon beta, glatiramer acetate, or laquinimod, all agents used to reduce the severity of MS, BDNF levels increase (Chen and Dhib-Jalbut, 2003; Caggiula et al., 2006; Bruck and Wegner, 2011). The evidence suggests that BDNF may have a neuroprotective role. Our results contribute to this possibility and suggest that it does so by enhancing the proliferation of OPCs that may influence the recovery from a demyelinating lesion.

## Summary

In response to demyelination, there are increases in oligodendrocyte progenitors that are reduced in mice deficient in BDNF. The present work indicates that this reduction is due to effects on DNA synthesis and subsequent cell proliferation but not differential actions on cell death.
